# Seasonality of birth in children with central nervous system tumours in Denmark, 1970–2003

**DOI:** 10.1038/sj.bjc.6604813

**Published:** 2008-12-09

**Authors:** L S Schmidt, K Grell, K Frederiksen, C Johansen, K Schmiegelow, J Schüz

**Affiliations:** 1 Institute of Cancer Epidemiology, Danish Cancer Society, Strandboulevarden 49, DK-2100 Copenhagen Ø, Denmark; 2 Department of Paediatrics, Copenhagen University Hospital, Denmark; 3 The Medical Faculty, University of Copenhagen, Copenhagen, Denmark

**Keywords:** brain tumour, central nervous system tumour, seasonal variation, birth, ependymoma, risk factor

## Abstract

We investigated possible seasonal variation of births among children <20 years with a central nervous system tumour in Denmark (*N*=1640), comparing them with 2 582 714 children born between 1970 and 2003. No such variation was seen overall, but ependymoma showed seasonal variation.

Tumours of the central nervous system (CNS) are the second most frequent malignancy in childhood and the leading cause of death from childhood cancer (Little, 1999). The aetiology is in most cases unknown, and likely to differ among the different morphological subgroups. Approximately 5% are associated with genetic syndromes such as neurofibromatosis and tuberous sclerosis (Little, 1999). The only well-established environmental risk factor is ionising radiation (Wakeford, 2004).

A few studies have reported marked and significant seasonality of birth among patients with a CNS tumour (Yamakawa *et al*, 1982; Heuch *et al*, 1998; Feltbower *et al*, 2001; McNally *et al*, 2002; Brenner *et al*, 2004; Halperin *et al*, 2004; Koch *et al*, 2006; Mainio *et al*, 2006; Hoffman *et al*, 2007). However, there is considerable variability across studies with respect to which histological subgroup showed the effect. Differences in study design and statistical methods may have contributed to the observed differences.

Seasonal variation in birth of patients diagnosed with a specific disease, which differs from the underlying seasonality of births in the general population, suggests an environmental factor operating at conception, during pregnancy or in the neonatal period. Such a seasonal pattern may reflect for example variation in exposure to sunlight or to infections or variation of the diet.

We used nationwide cancer and population registers to determine whether there is a seasonal pattern in the births of children who later develop a CNS tumour compared with the distribution of births of the general population in Denmark.

## Materials and methods

All children 0–19 years of age, who were diagnosed with a primary CNS tumour in the period 1970–2003 and registered in the Danish Cancer Registry, were included. Secondary tumours were excluded as treatment of a primary cancer is a known risk factor for secondary cancers (Maule *et al*, 2007). We included only tumours with a topography and morphology code defined in the International Childhood Cancer Classification second edition (ICCC-2; Kramarova and Stiller, 1996). In the period 1980–1996, all cases were validated using their medical records (Thorsteinsson *et al*, 2005).

The reference group included children born in 1970–2003, identified in the national Central Population Register (CPR) established in 1968 and subsequently used for all official personal registration of Danish citizens and residents. All live-born children are assigned a unique 10-digit CPR number, including the date of birth and gender code. A test for a sinusoidal variation was applied to evaluate changes of CNS tumour incidence by month of birth, adjusted for the background distribution of births (Walter and Elwood, 1975). The deviation of observed from expected numbers of cases was compared with a *χ*^2^-distribution with 2 degrees of freedom, and the timing of the peak was calculated. For comparison with a recent study (Hoffman *et al*, 2007), we defined the same subgroups by gender and age, young children aged 0–4 years at diagnosis and older children (5–19 years).

## Results

**Figure 1:**
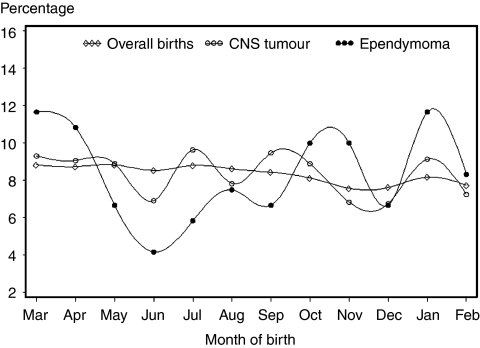
Distribution of births per month among patients with Central nervous system (CNS) tumours, ependymoma and among children in Denmark.

We identified 1640 eligible CNS tumour patients (Table 1) and the reference group comprised 2 582 714 children. Figure 1 illustrates the distributions of births in the general population, among patients with CNS tumours, and among ependymoma patients. Overall, no statistically significant evidence of seasonal variation of births was observed among patients with CNS tumour (Table 1). When stratifying by histology, we found a significant variation related to month of birth among children with ependymoma, with a peak in early January and a summer trough (Table 1). When examined by age and sex, ependymoma, showed a significant seasonal variation only at ages 5–19 years, and among girls (Table 1), but numbers in subgroups were small.

## Discussion

The major strength of our study is its base in high quality population-based administrative registers, ensuring complete case ascertainment and an appropriate unbiased reference group. The Walter and Edwood test may perform poorly when numbers are small (St Leger, 1976), as in our analyses by gender and age. If the aetiologically relevant exposure period is during pregnancy, then differences in gestational age at birth might dilute a possible seasonality effect. As the proportion of premature births (gestational age <37 weeks) only is 6% (The Danish National Board of Health, 2007), this is unlikely to have had a significant influence on our findings.

Examination of seasonal variation of birth in CNS tumours have had inconsistent results (Yamakawa *et al*, 1982; Heuch *et al*, 1998; Feltbower *et al*, 2001; McNally *et al*, 2002; Halperin *et al*, 2004; Hoffman *et al*, 2007), and overall, no seasonal pattern of birth is suggested. Seasonal variations sometimes seen for diagnostic subgroups were not consistent, so chance may have operated. Such random effects may also explain the significant seasonality for ependymomas in children aged 5–19 years and at all ages in girls, as no aetiological differences in the ependymomas are known in these subsets. As associations in the same direction were found for younger children and for boys, the absence of statistical significance may reflect a lack of power.

Notably, all previous positive findings regardless of subgroup have concerned a peak of births in the fall or winter, but most lacked an appropriate population-based comparison group.

Ependymoma showed seasonality in only one previous study (McNally *et al*, 2002), although the peak observed in February was not statistically significant (Edwards test *P*=0.10). Astrocytoma showed a seasonal pattern in two studies (Heuch *et al*, 1998; McNally *et al*, 2002), and medulloblastoma seasonality by month of birth in three (Yamakawa *et al*, 1982; Heuch *et al*, 1998; Hoffman *et al*, 2007).

Studies of the risk of adult brain tumours have, as with children, all found a peak of birth in winter (Brenner *et al*, 2004; Halperin *et al*, 2004; Koch *et al*, 2006; Mainio *et al*, 2006), although no studies from the southern hemisphere are available.

Dates of diagnosis showed seasonal variation and strong evidence of space-time clustering in the United Kingdom among children with astrocytoma and ependymoma, also supporting a role of infections in their aetiology (McNally *et al*, 2002); however, in Sweden clustering was not observed (Hjalmars *et al*, 1999). When interpreting these results one should keep in mind that the interval between the onset of disease and diagnosis is variable and often long because of a combination of unspecific or initially few symptoms, patients' delay, as well as doctors' delay.

Seasonal variation of birth is a fairly crude proxy measure for more specific exposures that vary seasonally around the time of conception, during pregnancy or in the neonatal period. It is unclear whether this seasonal variation of births in CNS tumour reflects variations in exposure to specific infections, variable levels of the burden of community infections or other factors. Some studies have suggested that exposure to infections of the mother or the child around the time of birth may be associated with increased risk of certain CNS tumours in children (Linet *et al*, 1996; Linos *et al*, 1998; Dickinson *et al*, 2002; McNally *et al*, 2002). In addition, three polyomaviruses have shown oncogenic properties when injected into the brain of newborn laboratory animals (White *et al*, 2005). Also seasonal changes in hours of sunlight per month, especially at Northern latitudes, subsequently leads to fluctuation in vitamin-D levels. Vitamin-D has antiproliferative and pro-apoptotic properties and may be involved in carcinogenesis (Holick, 2007). However, little is known about any role of D-vitamin in CNS tumours (Ko *et al*, 2005). Dietary habits may also be related to season, but relevant data for Denmark are lacking.

Little is known of ependymoma aetiology, partly because they only account for approximately 10% of CNS tumours in children, and most studies lack sufficient numbers. A considerable fraction of spinal ependymoma is associated with mutations in the neurofibromatosis type 2 gene (NF2; Ebert *et al*, 1999). An inverse association between maternal consumption of vitamin supplements during pregnancy and ependymoma risk has been observed (Bunin *et al*, 1993), whereas no association with birth weight has been reported (Harder *et al*, 2008).

As potential proxy exposures to infectious diseases, space-time clustering has been noted in ependymoma (McNally *et al*, 2002), and one study reported an association with a high number of siblings (Altieri *et al*, 2006).

In conclusion our results confirm the findings from most previous studies that no seasonal variation of births is apparent, when combining all histological subgroups of childhood CNS tumours. We cannot exclude that our finding of a seasonal pattern of births among patients with ependymoma is simply due to chance. But the fact that all studies which have reported a seasonal variation of birth, even though evident for different histological subgroups of CNS tumours, show an excess of births either during fall or during winter, justifies further analysis of the seasonality of births in large scale studies.

## Figures and Tables

**Table 1:** Evaluation of seasonality of birth by histology, gender and age

**Histology**		**Walter and Elwood's test**			
**ICCC-2**	**Cases**	***χ* ^2^**	***P*-value**	**Theta (*θ*)**	**Peak-month**
Ependymoma	162	8.0	0.02	13.81	January
					
Astrocytoma	607	1.45	0.48		
PNET	270	0.61	0.74		
Other glioma	76	3.28	0.19		
Other specified intracranial and intraspinal neoplasm	199	1.63	0.44		
Unspecified intracranial and intraspinal neoplasm	326	0.85	0.65		
All CNS tumours	1640	0.37	0.83		
					
*Ependymoma by age and gender*					
0–4 years	69	2.2	0.34		
5–19 years	93	10.3	0.006	−4.30	December
Boys	88	4.0	0.13		
Girls	74	9.7	0.008	−13.78	December

CNS=central nervous system; ICCC-2=International Childhood Cancer Classification second edition; PNET=primitive neuroectodermal tumour.
